# Crosstalk between endoplasmic reticulum and cytosolic unfolded protein response in tomato

**DOI:** 10.1007/s12192-022-01316-7

**Published:** 2022-11-30

**Authors:** Karin Löchli, Emma Torbica, Misgana Haile-Weldeslasie, Deborah Baku, Aatika Aziz, Daniela Bublak, Sotirios Fragkostefanakis

**Affiliations:** Molecular and Cell Biology of Plants, Goethe University Frankfurt, Frankfurt, D-60438 Germany

**Keywords:** Unfolded protein response, Heat stress transcription factors, Heat shock proteins, bZIP, Endoplasmic reticulum, Autophagy

## Abstract

Conditions that cause proteotoxicity like high temperature trigger the activation of unfolded protein response (UPR). The cytosolic (CPR) and endoplasmic reticulum (ER) UPR rely on heat stress transcription factor (HSF) and two members of the basic leucine zipper (bZIP) gene family, respectively. In tomato, HsfA1a is the master regulator of CPR. Here, we identified the core players of tomato ER-UPR including the two central transcriptional regulators, namely bZIP28 and bZIP60. Interestingly, the induction of ER-UPR genes and the activation of bZIP60 are altered in transgenic plants where HsfA1a is either overexpressed (A1aOE) or suppressed (A1CS), indicating an interplay between CPR and ER-UPR systems. Several ER-UPR genes are differentially expressed in the HsfA1a transgenic lines either exposed to heat stress or to the ER stress elicitor tunicamycin (TUN). The ectopic expression of HsfA1a is associated with higher tolerance against TUN. On the example of the ER-resident Hsp70 chaperone BIP3, we show that the presence of *cis*-elements required for HSF and bZIP regulation serves as a putative platform for the co-regulation of these genes by both CPR and ER-UPR mechanisms, in the case of BIP3 in a stimulatory manner under high temperatures. In addition, we show that the accumulation of HsfA1a results in higher levels of three ATG genes and a more sensitized induction of autophagy in response to ER stress which also supports the increased tolerance to ER stress of the A1aOE line. These findings provide a basis for the coordination of protein homeostasis in different cellular compartments under stress conditions.

**Supplementary Information:**

The online version contains supplementary material available at 10.1007/s12192-022-01316-7.

## Introduction

Protein homeostasis is a prerequisite for the optimal growth and development of all organisms. Exposure of plants to environmental cues such as heat stress (HS), drought, or salinity can cause proteotoxicity due to the accumulation of misfolded proteins that can eventually lead to cell death. Molecular chaperones such as heat shock proteins (HSPs) are essential for maintenance of protein homeostasis by assisting protein quality control, folding, and sorting of proteins to the designated cellular compartments (Vierling 1991).

Proteostasis in the endoplasmic reticulum in plants is mainly regulated by membrane-associated transcription factors belonging to the bZIP gene family (Liu and Howell 2016). Two UPR branches have been discovered so far in plants, with one arm involving bZIP28 and the other one both the bZIP60 and the kinase/ribonuclease IRE1 (Liu et al. 2007a, b; Iwata et al. 2008; Ruberti and Brandizzi 2014). Under non-stress conditions, binding of the ER-resident HSP70 chaperone BIP to the luminal domain of bZIP28 leads to the sequestration of the latter to the ER (Sun et al. 2013). Accumulation of unfolded proteins competes for BIP which leads to its dissociation from bZIP28. bZIP28 is transported to Golgi where metalloproteases site 1 protease and site 2 protease (S1P and S2P) cleave the protein to release the cytosolic domain which relocates to the nucleus to bind to *cis*-elements of ER-UPR-related genes (Liu et al. 2007b; Gao et al. 2008). bZIP28 functions in association with NF-Y factors on promoters containing the ER stress element (ERSE-I: CACG-N10-CCAAT) where the bZIP dimers bind to the core CACG element and NF-Y factors to CCAAT (Liu and Howell 2010). bZIP17 in *Arabidopsis* *thaliana* is closely related to bZIP28 and operates under salt stress conditions (Liu et al. 2007b).

The accumulation of unfolded proteins also leads to the oligomerization of IRE1, a membrane-tethered protein with a C-terminal luminal domain and an N-terminal region containing a kinase and a ribonuclease domain (Nagashima et al. 2011; Hayashi et al. 2012). Oligomerization of IRE1 leads to the activation of the RNase domain which cleaves the mRNA of bZIP60 (Deng et al. 2011; Hayashi et al. 2012). The unspliced bZIP60 mRNA is translated to a protein containing a bZIP domain and a TMD (Iwata et al. 2008). Splicing due to IRE1 is based on the recognition of a conserved double stem-loop structure which is partially cleaved (Deng et al. 2011). Ligation of the 5′ and 3′ mRNA parts leads results into spliced bZIP60 (bZIP60s) mRNA that due to a frameshift that is translated to a protein lacking the TMD. bZIP60s translocates to the nucleus to bind to *cis*-elements in promoters of ER-UPR genes. UPRE-III (TCATCG) is a binding site for AtbZIP60 on the promoter of NAC103 (Sun et al. 2013) while UPRE-II (GATGACGCGTAC) is a binding site for rice bZIP60 orthologue (Hayashi et al. 2013). bZIP60 but also bZIP28 can bind to plant UPRE (P-UPRE: ATTGGTCCACGTCATC) found in promoters of ER stress–responsive genes (Iwata and Koizumi 2005; Tajima et al. 2008).

bZIP28 and bZIP60 can heterodimerize and have been shown to share common targets albeit specific downstream genes exist as well (Liu and Howell 2010; Ruberti et al. 2018). ER-resident BIP and Hsp90, as well as members of the folding apparatus such as calnexin (CNX) and calreticulin (CRT) coding genes, are induced upon ER stress (Iwata et al. 2010). ER-resident small HSPs (ER-sHSP) are the only class of chaperones that has not been attributed as part of the ER-UPR as at least in *A. thaliana* the single ER-sHSP gene is neither induced by ER stress elicitors and nor regulated by bZIP60 or bZIP28. However, overexpression of tomato Hsp21.5 enhanced the resistance of young tomato seedlings enhanced to tunicamycin (TUN), a glycosylation inhibitor and ER-UPR inducer (Zhao et al. 2007). ER-resident sHSP proteins are considered to be part of the heat stress transcription factor (HSF)–dependent network (Scharf et al. 2001; Liu and Charng 2013; Fragkostefanakis et al. 2015) while tomato Hsp21.5A is regulated by WHIRLY1 (Zhuang et al. 2020).

The induction of cytosolic HSPs is regulated by members of the large gene family of HSFs (Scharf et al. 2012; Ohama et al. 2017). HSFs bind to repetitive palindromic motifs called HS elements, 5′-GAAnnTTC-3′ typically located in the near vicinity upstream of the TATA box in eukaryotic HS-induced genes (Treuter et al. 1993). Functional HSEs for HSF binding require the invariable G and C residues with an A residue in either position + 2 or + 3, and a T residue in position + 6 or + 7 (Santoro et al. 1998; Scharf et al. 2001). Independent single HSEs are ubiquitously found in gene promoters (Fragkostefanakis et al. 2015) but typically more than two consecutive motifs are required for HSF-binding oligomers (Treuter et al. 1993). All major stress-induced cytosolic HSPs have HSEs in their promoters and therefore the activation of HSFs is essential for their induction under stress conditions (Scharf et al. 2001; Fragkostefanakis et al. 2015).

While both the CPR and ER-UPR are activated under HS and are important for thermotolerance, they were initially considered to act at large independently (Sugio et al. 2009). Only recently, it was shown that the induction of maize HsfA6b/HSF13 under HS is in part dependent on bZIP60 marking the first direct evidence for the existence of a crosstalk between ER and cytosolic UPR (Li et al. 2020). Previously, it was proposed that some genes such as ERDJ3a might be regulated by both bZIP60/bZIP28 and HSFs as they contain UPRE and HSE (Howell 2017). In addition, the heat stress induction of bZIP28 as well as BIP1/2 genes was affected in a negative and positive manner in the HsfA1 quadruple *A. thaliana* mutant (Liu and Charng 2012), while two BIP proteins were found to accumulate at higher levels in tomato HsfB1 overexpression and suppression lines (Fragkostefanakis et al. 2018).

Another level of a possible crosstalk between CPR and ER-UPR is the regulation of autophagy. Autophagy is induced by adverse environmental conditions as well as by ER stress elicitors such as TUN (Srivastava et al. 2018). It is important for the degradation of protein aggregates and recycling (Liu et al. 2012; Liu and Bassham 2013). In Arabidopsis, the induction of autophagy is dependent on the activation of IRE1b via the IRE1-dependent decay of messenger RNA (RIDD) mechanism which mediates the mRNAs that code for secreted proteins, thereby reducing ER protein load (Bao et al. 2018). Autophagy is linked to the proteotoxicity levels as it can be induced by the expression of a misfolded protein but suppressed by the overexpression of BIP (Yang et al. 2016). Autophagy is also mediated by the activity of HSFs. ATG10 and ATG18f are regulated by tomato HsfA1a in response to drought, and ATG10 in tomato pollen heat stress response (Wang et al. 2015; Xie et al. 2022).

All these results suggest a crosstalk between HSR and UPR which at the moment is not well understood. We addressed this question using tomato which has a well-defined HSF system with only a single gene, HsfA1a, acting as master regulator of heat stress response (Mishra et al. 2002). We show that tomato seedlings ectopically expressing HsfA1a have a higher tolerance against the ER stress elicitor TUN, due to the stronger induction of ER chaperone coding genes. Surprisingly, the accumulation of HsfA1a is associated with the increased accumulation of the spliced bZIP60 (bZIP60s) transcripts, highlighting the central role of HSF system for the coordination of the cellular stress response branches under elevated temperatures.

## Materials and methods

### Plant material and stress treatments

Seedlings of *Solanum lycopersicum* cv. Moneymaker of either wild type (WT) or transgenic HsfA1a overexpression (A1aOE) and suppression lines (A1CS) described earlier (Mishra et al. 2002) were grown on Gelrite-solidified Murashige and Skoog (MS) medium (Murashige and Skoog 1962) supplemented with 20 g L^−1^ sucrose. ER stress was induced by subjecting the whole young seedlings to either tunicamycin (Sigma-Aldrich, T7765) at the indicated concentrations for each experiment or DMSO (0.1%). The IRE1 inhibitor 4μ8c (Sigma-Aldrich, SML0949) was supplemented at a final concentration of 0.25 μM, the 3-MA (Sigma-Aldrich, M9281) at 5 mM, and Torin2 (Sigma-Aldrich, SML1224) at 2 μΜ. For heat stress treatments, seedlings or detached leaves from 6- to 8-week-old plants were exposed at the indicated regimes for each experiment sealed in Petri dishes on wet paper towels in water baths.

### Gene expression analysis

Total RNA was extracted using the TRIzol reagent (Invitrogen) according to the manufacturer’s instructions. cDNA was synthesized with the reverse transcriptase RevertAid (Thermo Fisher) following the instruction of the manufacturer. The transcript levels of genes in cDNA samples were determined by quantitative real-time PCR (qRT-PCR) on a StepOnePlus cycler (Thermo Fisher). qRT-PCR reaction was done using PowerUp SYBR mix (Thermo Fisher) with 0.3 μM of each oligonucleotide. Thermal cycling conditions were 50 °C for 2 min, followed by 95 °C/3 min, and then 40 cycles of 95 °C/15 s, 60 °C/30 s, and 72 °C/30 s. Oligonucleotides (Supplementary Table 1) were designed using PRIMER3 (www.genome.wi.mit.edu/cgi-bin/primer/primer3.cgi/). Oligonucleotide sequences for ATG genes were taken from Wang et al. (2015) and HsfA1a, HsfA2, and Hsp17.7A-CI from El-Shershaby et al. (2019). All qRT-PCR reactions were performed in triplicates and experiments were conducted three independent times. Relative transcript levels were calculated with the mean values of technical replicates according to the 2^−ΔΔCt^ method using EF1a (Solyc06g005060) as reference gene (Livak and Schmittgen 2001).

### Protoplast isolation and transient expression assays

Tomato mesophyll protoplasts were isolated and transformed with plasmid DNA by polyethylene glycol (PEG)–mediated transformation (Mishra et al. 2002). Briefly, 10 µg of plasmid DNA was used to transfect 50,000 protoplasts. pRT-Neo plasmid carrying a neomycin phosphotransferase gene was used as mock control. For protein expression and GUS reporter assays, protoplasts were incubated for approximately 4–6 h at 25 °C. Relative GUS activity was measured as previously described (Treuter et al. 1993).

### MDC staining and microscopy

Monodansylcadaverine (MDC) staining was used to detect autophagosomes in protoplasts. Young leaves of 3-week-old tomato plants (WT, A1CS, and A1aOE lines) cultivated in soil under standard conditions (16 h light 25 °C/8 h dark 22 °C) were used for protoplast isolation. Protoplasts were treated with TUN or DMSO as control in K3M buffer for 3 h and then with 0.05 mM MDC (Sigma-Aldrich, 30,432) for 5 min in the dark as previously described (Bao et al. 2018). MDC stained autophagosomes were detected after excitation at 405 nm and emission at 556 nm wavelength under a Leica SP5 confocal laser scanning microscope (CLSM). Intracellular localization of bZIP28 was determined by detection of GFP (488 nm/490–548 nm) signal under a CLSM. Chlorophyll autofluorescence was measured at 665–738 nm.

### Immunoblot analysis

Protein extraction was done from homogenized tissues, re-suspended in 2 × volumes of high salt buffer (HSB) (Tillmann et al. 2015). Protein extraction from protoplasts was done after resuspension of cells to 60 μl HSB (Hahn et al. 2011). The samples were subsequently centrifuged for 5 min at 14,000 rpm and 4 °C. The supernatant was supplemented with 4 × SDS loading buffer and boiled at 95 °C for 5 min. Extracts were separated on 10 or 12% SDS–polyacrylamide gels. For immunoblot analysis, proteins were transferred to a nitrocellulose membrane (Protran nitrocellulose transfer membrane; Whatman) and protein signals were detected using chemiluminescence following the manufacturer’s protocol (Western Lightning Plus ECL solutions; Perkin-Elmer). The antibodies used were GFP (Roche), hemagglutinin tag (HA; Covance), and actin (Sigma-Aldrich). Antibodies used for detection of HsfA1a have been previously described (Lyck et al. 1997). Immunosignals were visualized with horseradish peroxidase (HRP)–coupled secondary antibodies (Sigma-Aldrich) and recorded in a ChemoStar ECL imager (INTAS, Göttingen, Germany).

### Expression and GUS reporter constructs

bZIP60s and bZIP28p wild type and mutants were cloned in pRT vector. bZIP60s was cloned in the C-terminus of a 3xHA-tag (pRT-CaMV35S::3xHA-bZIP60s) with SalI and BcuI restriction enzymes after PCR amplification. bZIP28 wild type or truncated mutant was cloned in the N-terminus of a 3xHA using Acc65I and EcoRI restriction enzymes. Deletion mutants were generated by PCR as described previously (Liu and Naismith 2008). The truncated bZIP28p(ΔN) was fused to the C-terminus of GFP via Acc65I and BcuI restriction enzymes (pRT-CaMV35S::3xHA-bZIP28p(ΔN)). The promoters of GUS reporter constructs contained an approximately 1000 bp region upstream of the TSS as indicated by SGN database (solgenomics.net). Inserts were amplified by PCR to introduce restriction sites and cloned in the pRT-GUS vector by T4 ligase cloning. All primers used for cloning as well as the restriction enzymes are shown in Supplementary Table 2.

### In silico promoter analysis

For promoter analysis, approximately 1000 bp DNA region upstream of the transcriptional unit was used for *cis*-element identification via PlantPAN v3.0 (Chow et al. 2019) using *A. thaliana* database. Heat shock elements (HSEs) were identified using the manual motif search with three consecutive palindromic nGANn or nGNAn on either + or − strand (Scharf et al. 2001). As ER-UPR-related *cis*-elements, we searched for pERSE: CCAAT-N10-CACG; ERSE-II: ATTGGNCCACG; pUPRE: ATTGGTCCACGTCATC; UPRE-I: CAGCGTG; UPRE-II: TACGTG; UPRE-III: TCATCG; and the motifs corresponding to binding sites for *A. thaliana* ER-UPR-related transcription factors bZIP17, bZIP28, and bZIP60: TFmatrixID_0027, TFmatrixID_0028, TFmatrixID_0189.

### Statistical analysis

All experiments were performed at least three times. Statistical analysis was performed using SPPS (v21) with either ANOVA and post hoc Duncan’s multiple range test (*p* < 0.05) or *t*-test.

## Results

### Regulators of ER-UPR in tomato

**Fig. 1:**
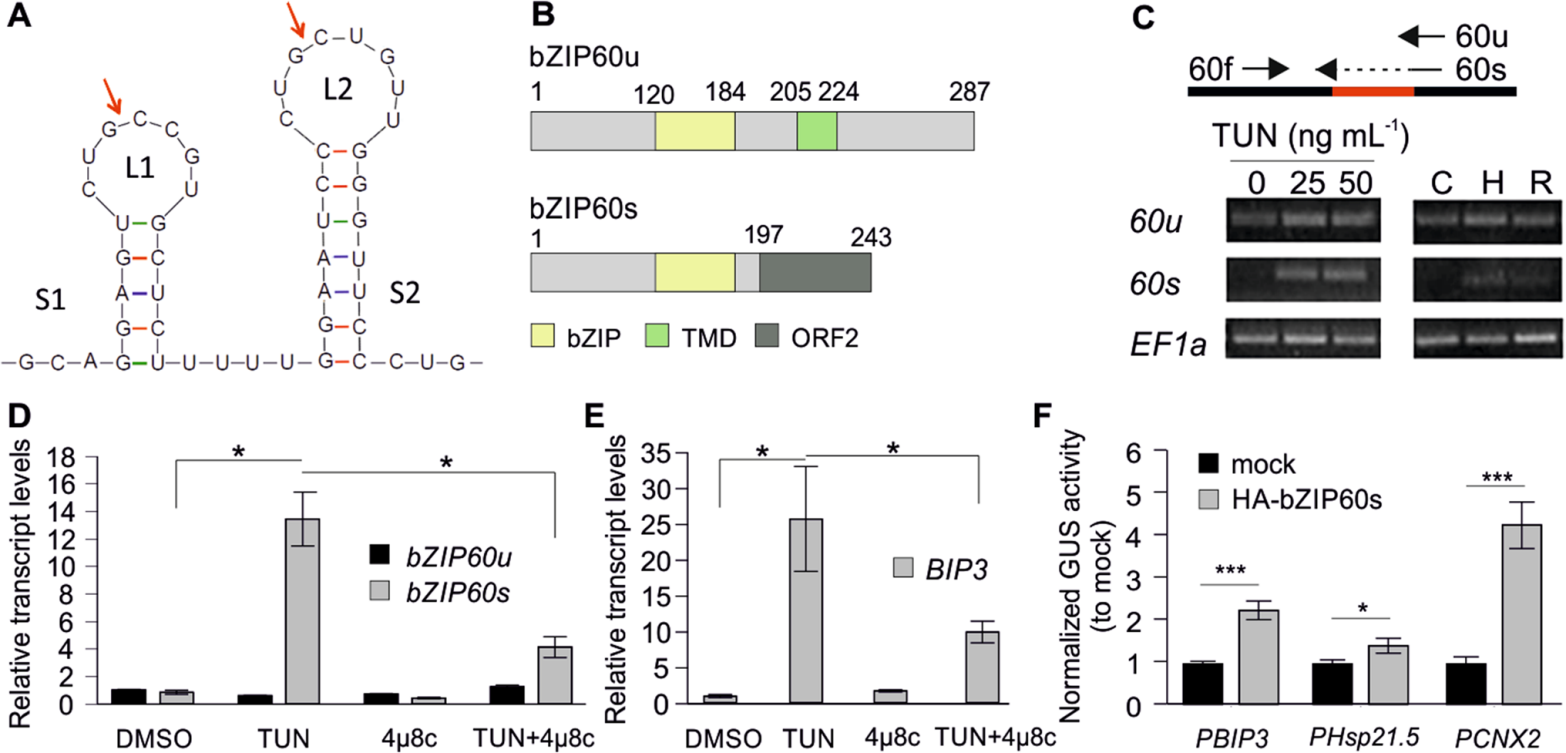
Tomato bZIP60 regulation and transactivation activity. **A** Double stem (S)-loop (L) structure of the *bZIP60u* mRNA recognized by IRE1 as predicted by RNAFold (Zuker and Stiegler 1981). Arrows indicate the cleavage sites. **B** Domain structure of bZIP60u and bZIP60s. Numbers indicate amino acid positions. bZIP, basic leucine zipper; TMD, transmembrane domain; ORF2, open reading frame generated by the frame shift. **C** RT-PCR of bZIP60u (unspliced) using 60f/60u oligonucleotides and bZIP60s (spliced) using 60f/60 s oligonucleotides, in seedlings exposed to DMSO (0 ng mL^−1^), 25 or 50 ng mL^−1^ of TUN. Seedlings were also exposed to a heat stress regime: 25 °C as control (C), 40 °C for 1 h (H), and then allowed to recover at 25 °C for 1.5 h (R). *EF1a* is the housekeeping gene. Relative transcript levels of **D** *bZIP60u* and *bZIP60s* or **E** *BIP3* in young tomato leaves treated with DMSO or TUN (50 ng mL^−1^) and/or 4μ8c (0.25 µM). Relative transcript levels were determined by qRT-PCR using 2^−ΔΔ^.^Ct^ method, *EF1a* as reference gene and normalized against DMSO WT as control sample. Each value is the average of three independent biological replicates. Error bars are ± SEM and asterisks depict significant differences based on ANOVA with Duncan’s multiple range test (*p < 0.05) for each gene. **F** Transactivation activity of bZIP60s using GUS reporter assay on promoters (1 kb) of selected tomato genes. Values are average of normalized GUS activity relative to mock control (*n* = 3). Error bars are ± SEM and asterisks indicate significant differences based on Student’s *t*-test (*p < 0.05, ***p < 0.001)

We first aimed to characterize the main regulators of ER-UPR in tomato. The orthologues of *A. thaliana* bZIP60 and bZIP28 are coded by tomato genes Solyc10g078290 and Solyc04g082890, respectively (Fragkostefanakis et al. 2015). Based on sequence similarity with *A. thaliana* bZIP60 and previous reports, we were able to confirm that the putative membrane-bound inactive tomato bZIP60u (unspliced) encodes for a 287 amino acid protein, predicted to have a transmembrane domain at the C-terminal region (Fig. 1A) and confirmed the conserved double stem-loop RNA structure that is recognized and cleaved by IRE1, leading to the deletion of 23 nt (Fig. 1B). We further confirmed the accumulation of the spliced bZIP60 transcripts (*bZIP60s*) by RT-PCR on leaves either exposed to increasing amounts of TUN or to HS (Fig. 1C). Sequencing of the bZIP60s transcript confirmed the spliced region as predicted (not shown).

The accumulation of *bZIP60s* as well as *BIP3*, a potent target of bZIP60s, in TUN-treated samples was reduced when samples were also treated with 4μ8C, a repressor of IRE1 endonuclease activity (Cross et al. 2012; Srivastava et al. 2018) (Fig. 1D, E). bZIP60s was cloned under CaMV 35S promoter and fused to the C-terminus of a triple HA to examine the transactivation activity of the factor on selected GUS reporter constructs. As we used the active bZIP60s, a stress is not required and therefore the assay was performed at 25 °C. Transient expression of the HA-tagged bZIP60s in protoplasts along with GUS reporter constructs carrying the promoter region of Hsp21.5, CNX2, and BIP3 resulted in increased GUS activity relative to the mock, confirming that the factor is active (Fig. 1F).

**Fig. 2:**
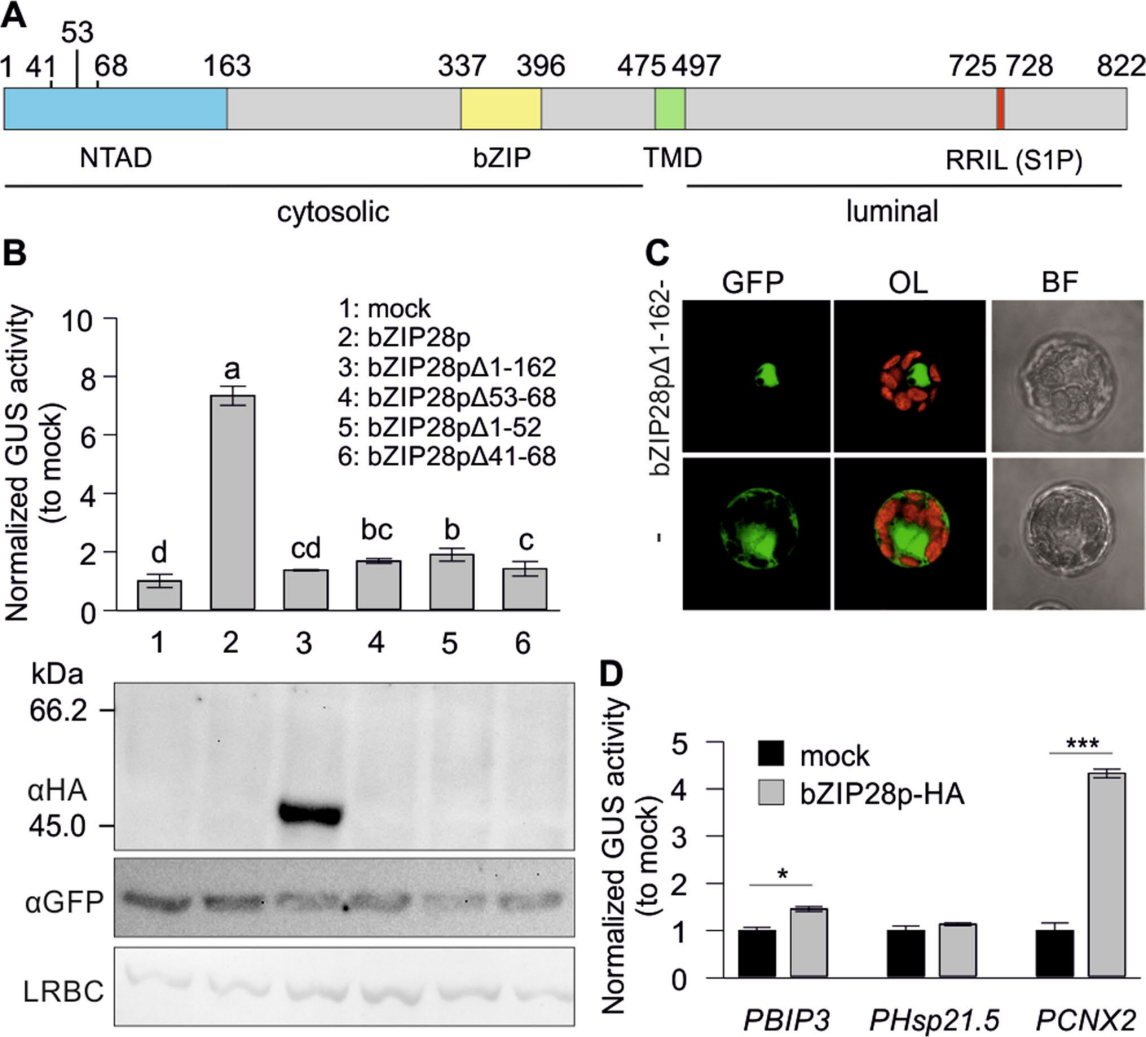
Tomato bZIP28 transactivation activity. **A** Schematic representation of the domains of bZIP28. Numbers indicate amino acids. NTAD, N-terminal activation domain region; bZIP, basic leucine zipper; TMD, transmembrane domain; S1P, metalloprotease recognition site. **B** GUS reporter assay on *PCNX1*::GUS reporter of the indicated bZIP28p mutants expressed in tomato mesophyll protoplasts. Values are average of normalized GUS activity relative to mock control (*n* = 3). Error bars are ± SD and different letters indicate significant differences based on ANOVA and Duncan’s multiple range test (*p* < 0.05). Immunoblot analysis of HA-tagged bZIP28p proteins expressed in protoplasts for the GUS reporter assay. LBRC, large subunit of RuBisCO. **C** Subcellular localization of bZIP28pΔ1-162-GFP or GFP alone in tomato protoplasts. Images are representative of many independent cells that showed the same result. OL, overlay of GFP and autofluorescence; BF, brightfield. **D** Transactivation activity of bZIP28p using GUS reporter assay on promoters (1 kb) of selected tomato genes. Values are average of normalized GUS activity relative to mock control (*n* = 3). Error bars are ± SEM and asterisks indicate significant differences based on Student’s *t*-test (*p < 0.05, ***p < 0.001)

The annotated Solyc04g082890 has a truncated N-terminal region compared to *A. thaliana* bZIP28 (Supplementary Fig. 1). Inclusion of the upstream start codon revealed that the annotated protein likely lacks the N-terminal 162 amino acids. The processed protein containing the cytosolic portion of bZIP28, named bZIP28p, is inactive as indicated by a GUS reporter assay (bZIP28pΔ1-162; Fig. 2B). The missing N-terminal region is important for transactivation activity but not nuclear translocation, as a GFP-tagged bZIP28pΔ1-162 expressed in tomato mesophyll protoplasts accumulated in the nucleus, in contrast to GFP alone which showed a nucleocytoplasmic distribution (Fig. 2C; Supplementary Fig. 2). The bZIP28p, with the missing 162 aa, is active as indicated by the GUS reporter assay using *PCNX2*::GUS reporter (Fig. 2B). Interestingly, while the truncated bZIP28p protein is detectable using an HA antibody at the N-terminus of the protein, the full length protein is not, indicating that it undergoes rapid degradation (Fig. 2B, immunoblot).

We assumed that a rapid degradation is an important regulatory mechanism to control the activity of the protein, as previously reported for other transcription factors (Muratani and Tansey 2003). Since the activation domain of bZIP28 has not been characterized so far in any plant species to the best of our knowledge, we did a series of deletions to examine their effect on the transactivation activity and the stability of the bZIP28p. Deletion of the sequence coding for the 1–68 aa abolished the activity of the protein suggesting that the activation domain of bZIP28 lies in this region. Two deletions (Δ41-68 and Δ53-68), within regions rich in Phe and Asp residues representing the VP16 acidic activation domain found in many transcription factors, also resulted in very low activity confirming the presence of the activation domain in this region (Fig. 2B; Supplementary Fig. 2). However, none of these mutations resulted in the stabilization of the protein, indicating a decoupling of the transactivation activity and protein turnover.

### Expression of ER chaperones and UPR genes under proteotoxic conditions

**Fig. 3:**
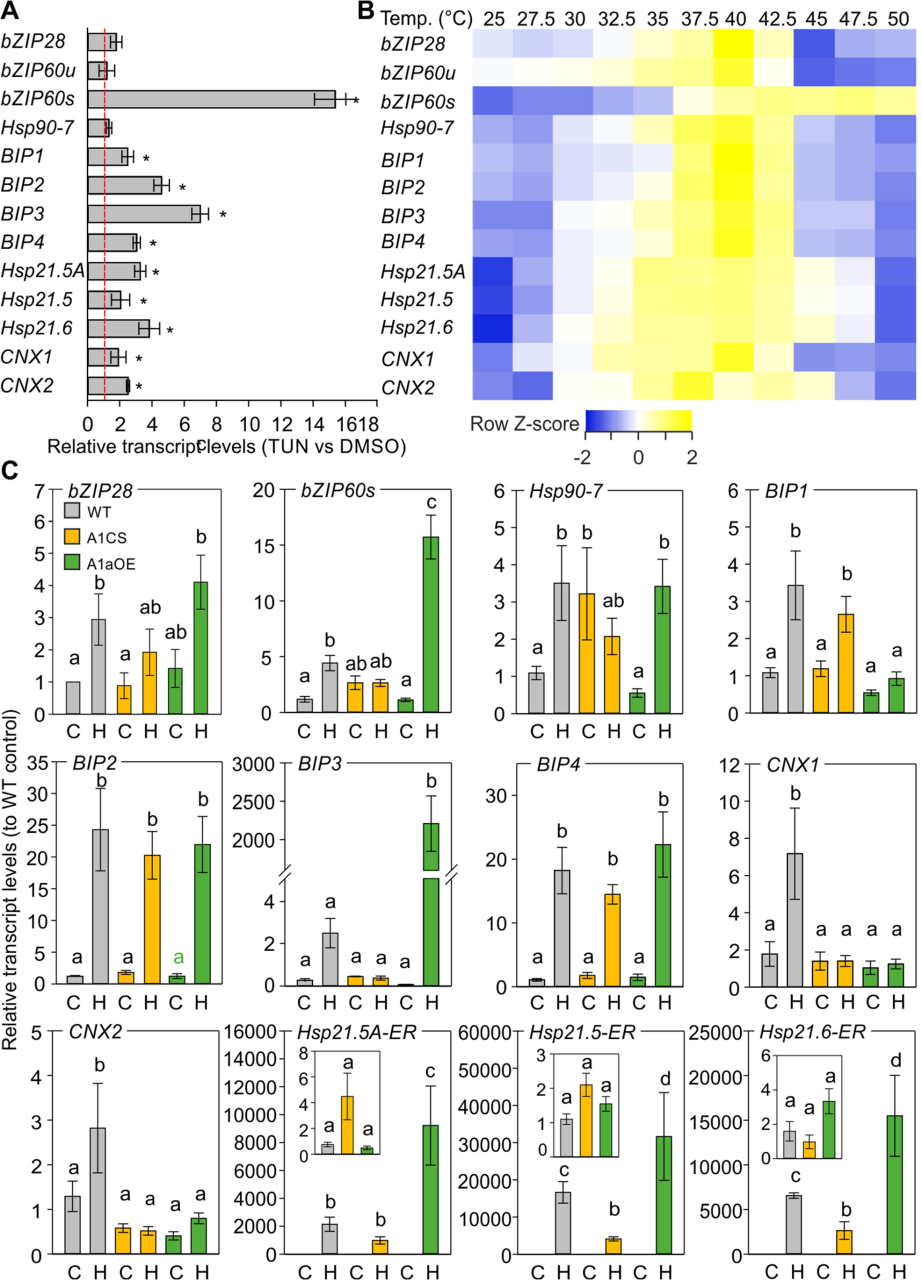
Transcript level of ER chaperones and UPR genes in response to tunicamycin or heat stress treatments. Relative transcript levels of selected genes in leaves treated with **A** TUN (50 ng mL^−1^) for 2 h or DMSO (control), **B** different temperatures for 1 h. Results are presented in **A** as 2^−ΔΔ^.^Ct^ or in **B** and **C** as − ΔΔCt. The asterisk indicates statistically significant difference based on Student’s *t*-test (*p* < 0.05). DMSO control onefold value is presented as a red line. **C** Relative transcript levels of ER-UPR genes in WT, A1CS, and A1aOE leaves exposed for 1 h at 40 °C (“H”) or 25 °C as control (“C”). Expression levels are expressed as relative to WT control. Bars are the average of three independent experiments, ± SEM. Different letters denote statistically significant differences based on one-way ANOVA with Duncan’s multiple range test (*p* < 0.05)

In a previous study, based on an orthology approach, we identified genes that code for ER chaperones and shown to be induced by ER stress elicitors in *A. thaliana*, collectively termed ER-UPR genes (Fragkostefanakis et al. 2015). Four BIP paralogues, three ER-resident sHSPs, and CNX-coding genes, as well as the spliced bZIP60 mRNA, accumulate in response to a 3-h TUN treatment (50 ng mL^−1^) on young tomato leaves compared to control samples treated with DMSO (0.1%) (Fig. 3A). In contrast, under these conditions, we did not observe any induction of bZIP28, Hsp90-7, or bZIP60u.

We further examined the responsiveness of some of these genes in seedlings exposed for 1 h to temperatures ranging from 25 to 50 °C (Fig. 3B). All genes showed a gradual increase in transcript levels that peaked at 40 °C and then reduced at higher temperatures to basal levels. Only *CNX2* showed a peak at 37.5 °C, while *bZIP60s* retained high levels at temperatures above 40 °C (Fig. 3B).

ER-UPR mediated mainly by bZIP60/bZIP28 and CPR controlled by HSFs is activated under high temperatures. To examine whether there is a crosstalk between CPR and ER-UPR, the transcript levels of ER-UPR genes were examined in transgenic lines that either HsfA1a is overexpressed (A1aOE) or suppressed (A1CS) (Mishra et al. 2002). HsfA1a shows an approximately 26-fold higher expression in A1aOE compared to WT, while co-suppression results in a 50% reduction of HsfA1a transcripts (data not shown). Nevertheless, as shown in Mishra et al. (2002), the co-suppression results in an almost complete absence of HsfA1a protein.

*bZIP60s* accumulated at significantly higher levels in A1aOE leaves when compared to WT, and so did *BIP3* and all three sHSPs (Fig. 3C). In contrast, all these genes showed significantly reduced levels in heat-stressed leaves of the A1CS transgenic line. Interestingly, the suppression of HsfA1a was associated with increased levels of Hsp90-7 in the control sample, while neither of the CNX coding genes was upregulated in response to HS in A1CS or A1aOE lines (Fig. 3C). These results indicate that the levels of HsfA1a and consequently the activity of the HSFs alter the transcript levels of genes coding for ER-resident chaperones and genes previously characterized as members of the ER-UPR network.

### Effect of HsfA1a overexpression or suppression on the tolerance of tomato seedlings to TUN

**Fig. 4:**
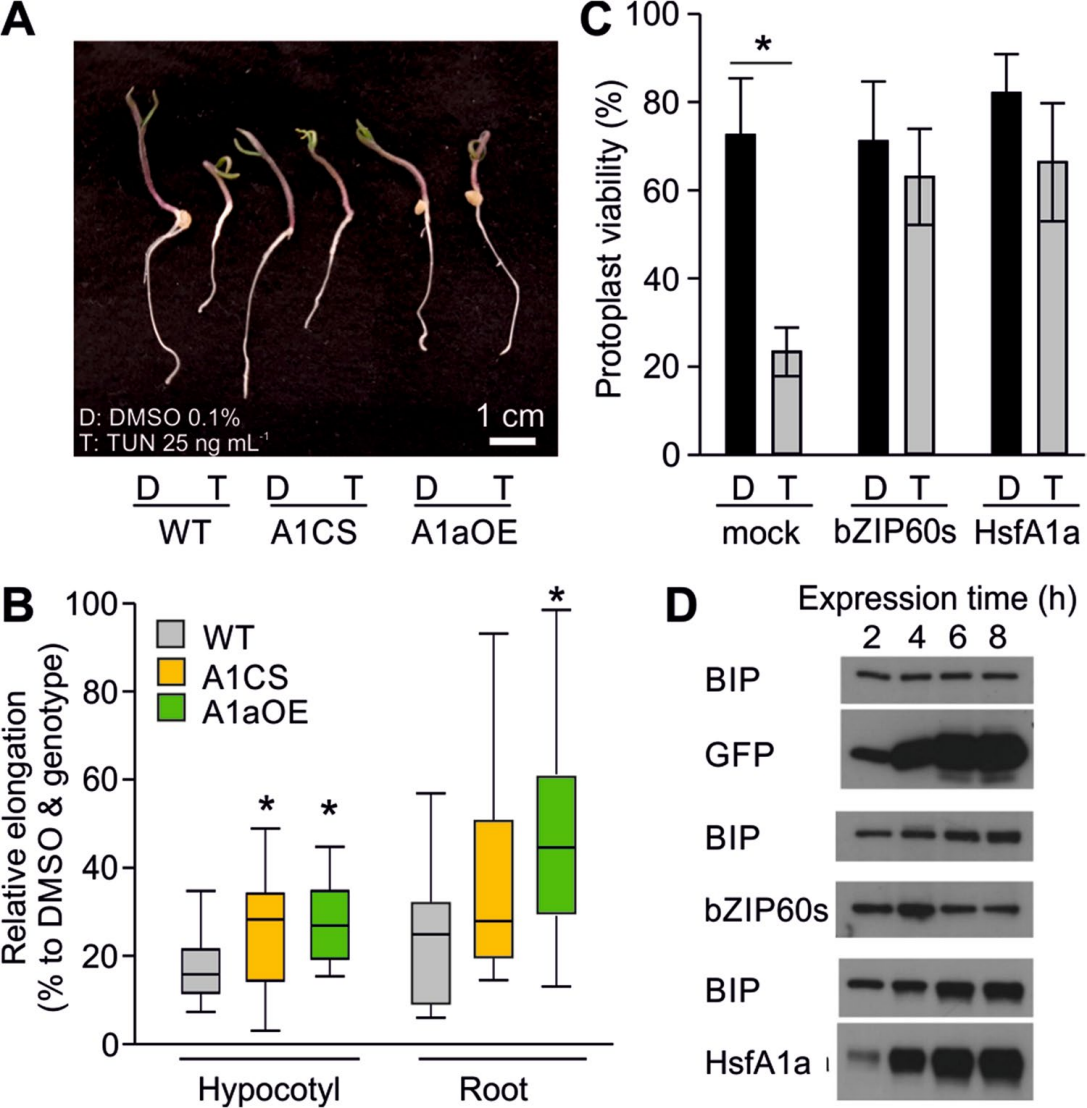
HsfA1a-dependent tolerance to tunicamycin. **A** Representative images of WT, A1CS, and A1aOE seedlings submerged in DMSO (0.1%) or TUN (25 ng mL^−1^) for 24 h and then allowed to recover for 3 days under standard conditions. **B** Relative hypocotyl and root elongation of seedlings exposed to TUN (25 ng mL^−1^) or DMSO for 24 h. Data shown as box plots derive from 20 to 25 seedlings from 3 biological replicates, and presented as percentages of growth of TUN relative to DMSO-treated seedlings for each genotype (DMSO is 100% for each genotype). **C** Percentage of viable over total number of mesophyll protoplasts expressing a mock plasmid, HA-bZIP60s or HsfA1a and treated either with DMSO or TUN (12.5 ng mL^−1^). **D** Immunoblot analysis showing the levels of endogenous BIP protein in protoplasts expressing GFP, HA-bZIP60s, or HsfA1a for the indicated time. The transgenes were detected using αGFP, αHA, and αHsfA1a, respectively

HsfA1a is a constitutively expressed gene mainly regulated at posttranslational level (El-shershaby et al. 2019; Mesihovic et al. 2022). We also confirmed that HsfA1a transcript levels do not significantly change in response to TUN treatment (Supplemental Fig. 3). However, the differential expression of ER-UPR genes in the HsfA1a transgenic lines suggests a potential crosstalk between CPR and ER-UPR. To address this question on the physiological level, we submerged young tomato seedlings from WT, A1CS, and A1aOE to TUN or DMSO for 24 h. Tolerance to TUN was determined as the hypocotyl and root elongation rates for each genotype during the recovery of the seedlings on MS medium normalized to control seedlings exposed for the same time to DMSO (Fig. 4A, B). WT seedlings showed a weak recovery in response to TUN. A1CS and A1aOE seedlings showed an increased hypocotyl relative recovery rate compared to WT. The elongation of the roots of A1aOE seedlings was significantly higher compared to WT or A1CS. These results collectively demonstrate that manipulation of HsfA1a levels affects the resilience of tomato seedlings to an ER stress elicitor.

The relation of HsfA1a activity with ER-UPR was further examined in mesophyll tomato protoplasts expressing bZIP60s-HA and HsfA1a or transfected with a mock plasmid (Fig. 4C). TUN reduced the viability of cells transfected with the mock plasmid by approximately 55%. In contrast, protoplasts expressing either bZIP60s or HsfA1a showed a mild non-significant reduction, suggesting that HsfA1a accumulation enhances the protection against ER stress (Fig. 4C). To check whether the enhanced tolerance is associated with increased chaperone levels in the ER, we followed the accumulation of the endogenous BIP proteins using an anti-BIP antibody in protoplasts expressing HsfA1a and compared to GFP or bZIP60s protoplasts serving as negative and positive controls, respectively (Fig. 4D). GFP expression while at very high levels did not have any effect on BIP levels. Interestingly, similar to bZIP60s-expressing cells, HsfA1a accumulation coincided with the accumulation of BIP (Fig. 4D).

### Regulation of ER-UPR genes and ER chaperones in HsfA1a transgenic lines in response to ER stress

**Fig. 5:**
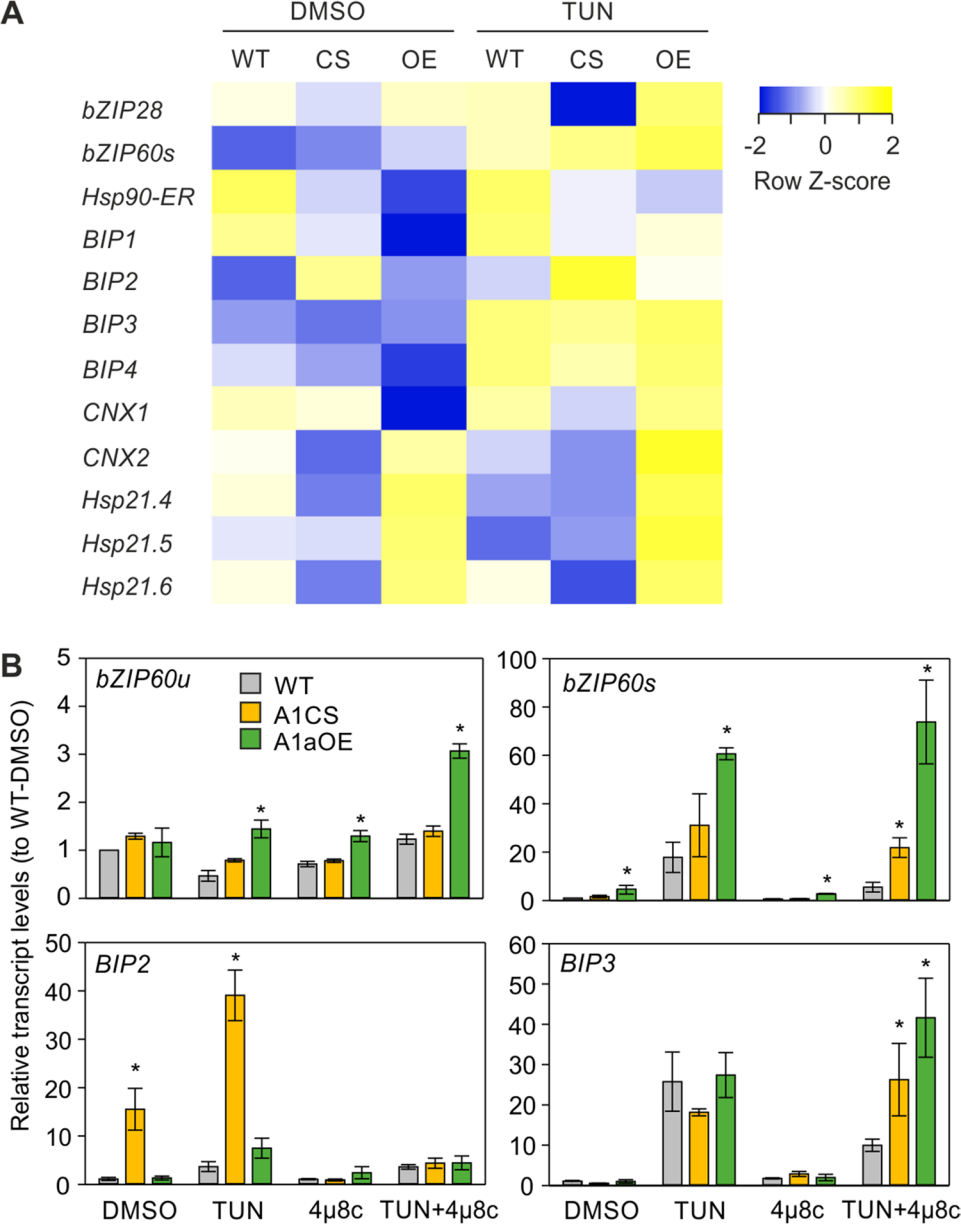
Effect of HsfA1a suppression or overexpression on ER-UPR. **A** Heat map of expression values of ER-UPR-related genes in WT, A1CS (CS), and A1aOE (OE) young tomato leaves treated with DMSO or TUN (50 ng mL^−1^) for 3 h. Relative transcript levels were determined by qRT-PCR using 2^−ΔΔ^^Ct^ method, *EF1a* as reference gene and normalized against DMSO WT as control sample. Each value is the average of three independent biological replicates. Values are shown as *Z*-score for each row. **B** Transcript levels of *bZIP60u*, *bZIP60s*, *BIP1*, and *BIP3* in tomato WT, A1CS, and A1aOE leaves treated with DMSO, TUN, and the IRE1 inhibitor 4μ8c. Each value is the average of three independent biological replicates and error bars are ± SEM. The asterisk denotes statistically significant difference between the indicated sample and the corresponding WT samples from the same treatment, based on *t*-test (*p* < 0.05)

The increased resilience of tomato seedlings, overexpressing or supressing HsfA1a against TUN, suggested a possible induction of ER-UPR components in these lines compared to WT. To examine whether the ectopic expression or suppression of HsfA1a in young tomato seedlings affects the expression of ER-UPR-related genes, we performed qRT-PCR on young leaves exposed to DMSO (0.1%) or TUN (50 ng mL^−1^) for 3 h (Fig. 5A). *BIP2* was significantly increased in A1CS leaves treated both with DMSO and TUN. *bZIP60s* and all three sHSPs accumulated at higher levels in DMSO-treated A1aOE samples, while *BIP3* and *CNX2* were also elevated in TUN-treated leaves of A1aOE line compared to WT (Fig. 5A).

To check whether the increased levels of *BIP2* and *BIP3* in A1CS and A1aOE lines respectively are due to the increased *bZIP60s* levels, we applied, in parallel to TUN, the IRE1 inhibitor 4μ8c (0.25 µM) (Fig. 5B). The inhibitor reduced the levels of *bZIP60s* in WT leaves treated with TUN, but had no significant effect on A1CS and A1aOE TUN-treated leaves. Interestingly, the levels of bZIP60u transcripts were significantly higher in all TUN and/or 4μ8c-treated samples when compared to the corresponding sample of WT leaves, indicating that HsfA1a overexpression induces the transcription of bZIP60 gene. 4μ8c suppressed the levels of *BIP2* in the A1CS line both in the presence and absence of TUN. In contrast, such an effect was not apparent for *BIP3* levels (Fig. 5B).

We also wondered whether CPR genes are affected by TUN treatment in the transgenic lines. Overexpression of HsfA1a resulted in higher accumulation of the Hsf-dependent *Hsp17.7A-CI*, while suppression in significantly lower levels of *HsfA2* (Supplemental Fig. 4). However, the levels of both genes were not affected by the TUN treatment.

### Transactivation activity of HSFs and ER-UPR-related bZIPs

**Fig. 6:**
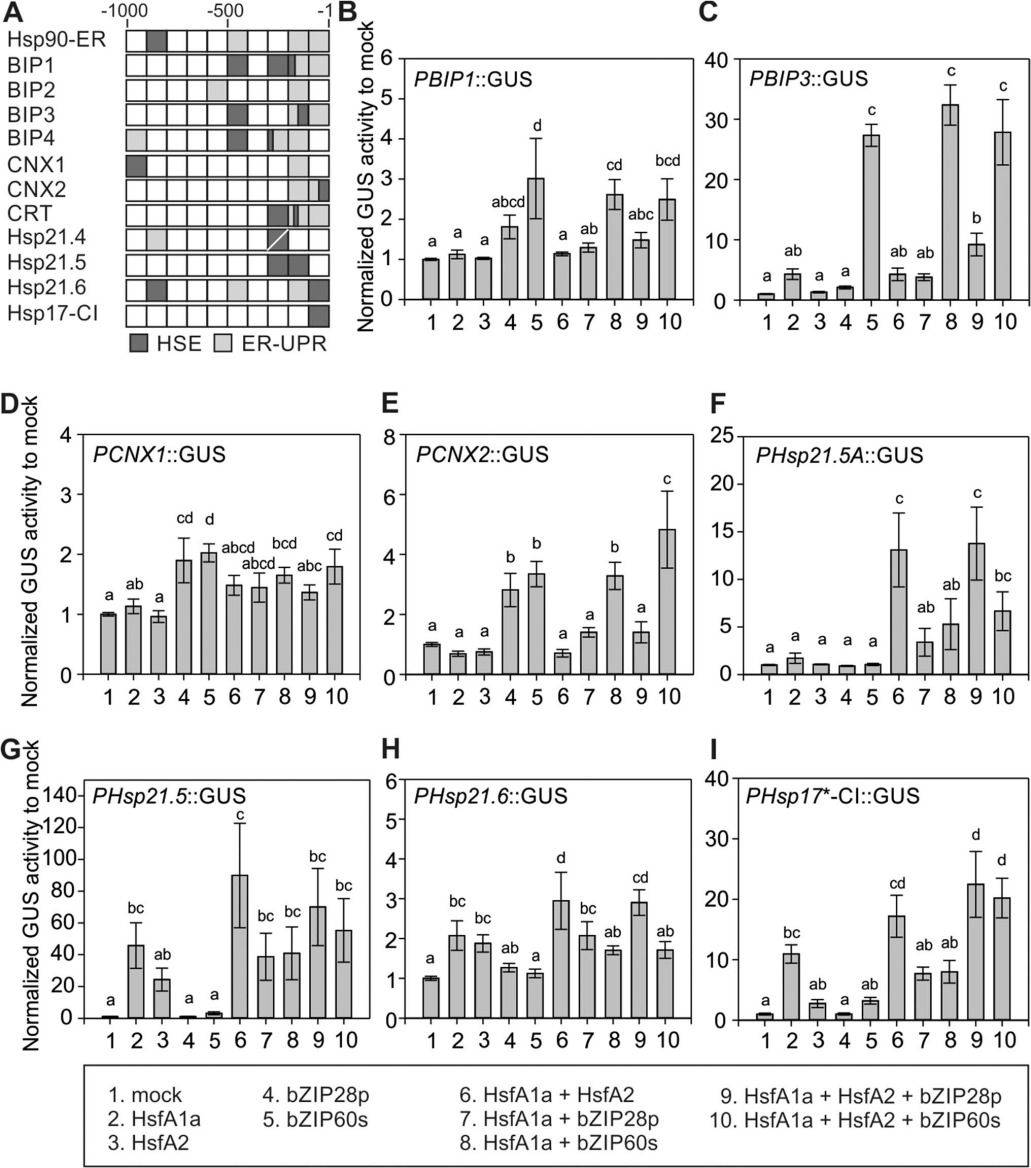
Transactivation activity of HsfA1a, HsfA2, bZIP60s, and bZIP28p on GUS reporters of ER chaperones and ER-UPR genes. **A** *Cis*-elements corresponding to HSF or bZIP28/bZIP60 binding sites in the promoters (approximately 1000 bp upstream of TSS) of ER chaperones and UPR genes. More detailed information can be found in Supplementary Dataset 1. **B**–**I** GUS reporter assays on different promoters in tomato protoplasts transformed with the indicated reporter and combinations of HsfA1a, HsfA2, bZIP28p, and/or bZIP60s. GUS activity values are the average of independent transformations from independent experiments (*n* = 6–9) and have been normalized against the mock control (onefold). Error bars are ± SEM. Different letters denote statistically significant difference between samples, based on one-way ANOVA test and Duncan’s multiple range post hoc test (*p* < 0.05)

The differential expression of ER-UPR genes in HsfA1a transgenic lines exposed to TUN or HS suggests a possible direct regulation of these genes by both ER-UPR-related bZIPs and HSFs. First, on a selected set of genes, we examined the presence of HSEs (three consecutive and palindromic nGANn or nGNAn: e.g., n**G**AAnnTT**C**nn**G**AAn) or ER-UPR elements (P-UPRE: ATTGGTCCACGTCATC; ERSE: CCAAT-N10-CACG; ERSE-II: ATTGG-N-CCACG; UPRE-III: TCATCG; TFmatrixID_0189: CACGT/ACGTG) (Fig. 6A; Supplementary Dataset 1). Through the in silico analysis, we were able to identify at least one type of ER-UPR element in each of the promoters (approximately 1000 bp) of ER chaperone coding genes, except *Hsp21.5* (Fig. 6A). Interestingly, we also found that HSEs are ubiquitous in these promoters as well, as all but *BIP2* contained at least one HSE. As previously reported, both ER-UPR elements and HSEs are more abundant within the 500 bp 3′-region of the promoter (Iwata et al. 2008).

The promoters of seven of these genes (BIP1, BIP3, CNX1, CNX2, Hsp21.5A, Hsp21.5, and Hsp21.6) were cloned to check the activity of bZIP and HSF factors via a GUS reporter assay (Fig. 6B–I). The activity of BIP and CNX reporter constructs was induced in protoplasts expressing either bZIP60s or bZIP28p. HSFs but not bZIPs induced the GUS activity of all three sHSP reporters (Fig. 6G–I).

The presence of both HSE and ER-UPR elements in these genes suggests possible co-regulation of transcription by both HSF and bZIP factors. In this direction, we co-expressed HsfA1a or HsfA1a/HsfA2 with either bZIP60s or bZIP28p and checked for possible significant differences in GUS activity when compared to sample expressing either bZIPs (in case of BIP, CNX) or HSFs (in case of sHSPs). Co-expression of either bZIP with HsfA1a or HsfA1a/HsfA2 did not result in any significant change of the GUS activity of any reporter construct when compared to the activity of the samples expressing of the single factors. Only the *PCNX2*::GUS reporter was induced at significantly higher levels in cells co-expressing HsfA1a/HsfA2/bZIP60s compared to all other combinations (Fig. 6E). Promoters lacking any apparent ER-UPR element such as Hsp21.5 or the PHsp17-CI having a minimal promoter containing only HSE are only controlled by HSFs and not bZIPs (Fig. 6G and I).

**Fig. 7:**
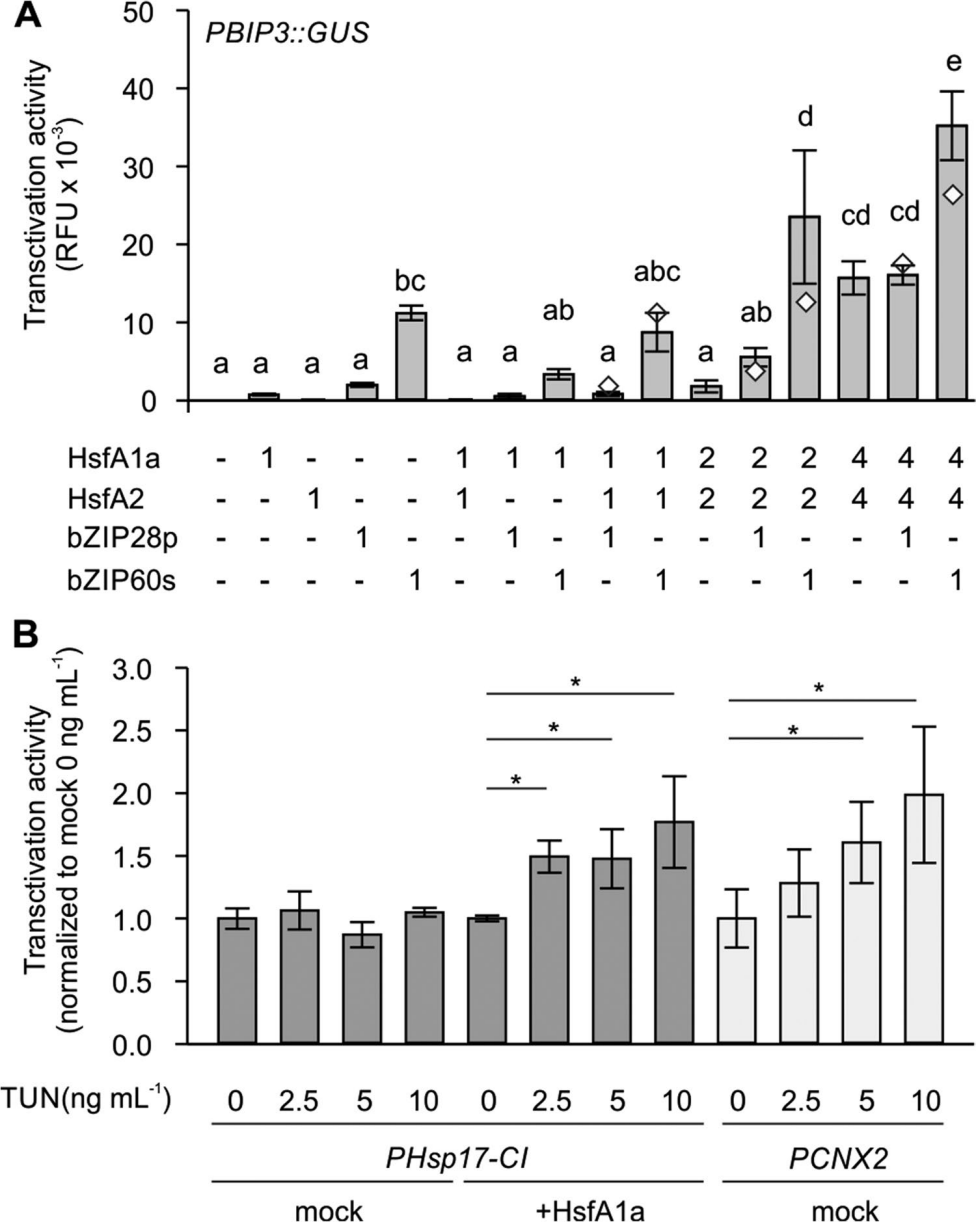
Transactivation activity of BIP3 GUS reporter by HSFs and bZIPs and TUN-triggered activity of HsfA1a. **A** GUS reporter assays of *PBIP3*::GUS in tomato protoplasts co-transformed with combinations of HsfA1a, HsfA2, bZIP28p, and/or bZIP60s. The amount of plasmid DNA carrying the transcription factor in micrograms used to transform 50,000 protoplasts is indicated below. GUS activity values are the average of independent protoplast transformations (*n* = 3). Error bars are ± SEM. Different letters denote statistically significant difference between samples, based on one-way ANOVA test and Duncan’s multiple range post hoc test (*p* < 0.05). **B** GUS reporter assay using either PHsp17-CI or CNX2 as reporters in protoplasts treated with the indicated amounts of TUN. GUS activity values are the average of independent transformations from independent experiments (*n* = 6–9) and have been normalized against the mock control (onefold) individually for each set. Error bars are ± SEM. Asterisks indicate statistically significant difference between samples, based on one-way ANOVA test and Duncan’s multiple range post hoc test (*p* < 0.05) performed independently for each set

Under HS conditions, *BIP3* showed a very strong induction in A1aOE line compared to WT (Fig. 3B), suggesting that the dramatic upregulation requires increased levels of HsfA1a. Transfection of protoplasts with 1 μg of plasmid carrying the expression cassette for HsfA1a, or 1 μg of each of HsfA1a and HsfA2 resulted in an only weak induction of BIP3 GUS reporter (Fig. 7A). We therefore co-transformed 2 × , or 4 × HsfA1a/HsfA2 with the BIP3 GUS reporter. Only the 4 × HsfA1a/HsfA2 combination yielded an induction of GUS activity. However, the combination of 2 × HsfA1a/HsfA2 with 1 μg bZIP28p showed a strong increase in GUS activity which exceeded the expected additive effect of the factors, suggesting a synergistic effect (Fig. 7A). A similar synergistic effect was detected when protoplasts were transformed with 4 × HsfA1a/HsfA2 and bZIP60s. These results suggest a possible direct, co-regulation of BIP3 by HSFs and bZIP28 and/or bZIP60.

We also asked, whether the TUN treatment affects the transactivation activity of HsfA1a. For this, a GUS reporter assay was used with the HSF-dependent reporter PHsp17-CI::GUS, or the bZIP28/bZIP60-dependent *PCNX2*::GUS (Fig. 7B). Tomato mesophyll protoplasts transfected with these reporters were treated with 2.5, 5, or 10 ng mL^−1^ of TUN. The treatment had no effect on Hsp17-CI reporter, but induced the GUS activity of the CNX2 reporter, confirming the validity of the stress induced by TUN. GUS activity for Hsp17-CI was induced in TUN-treated samples the presence of HsfA1a (Fig. 7B). These results indicate that the induction of ER stress can stimulate the transactivation activity of HsfA1a.

### Regulation of ER stress triggered autophagy by HsfA1a

**Fig. 8:**
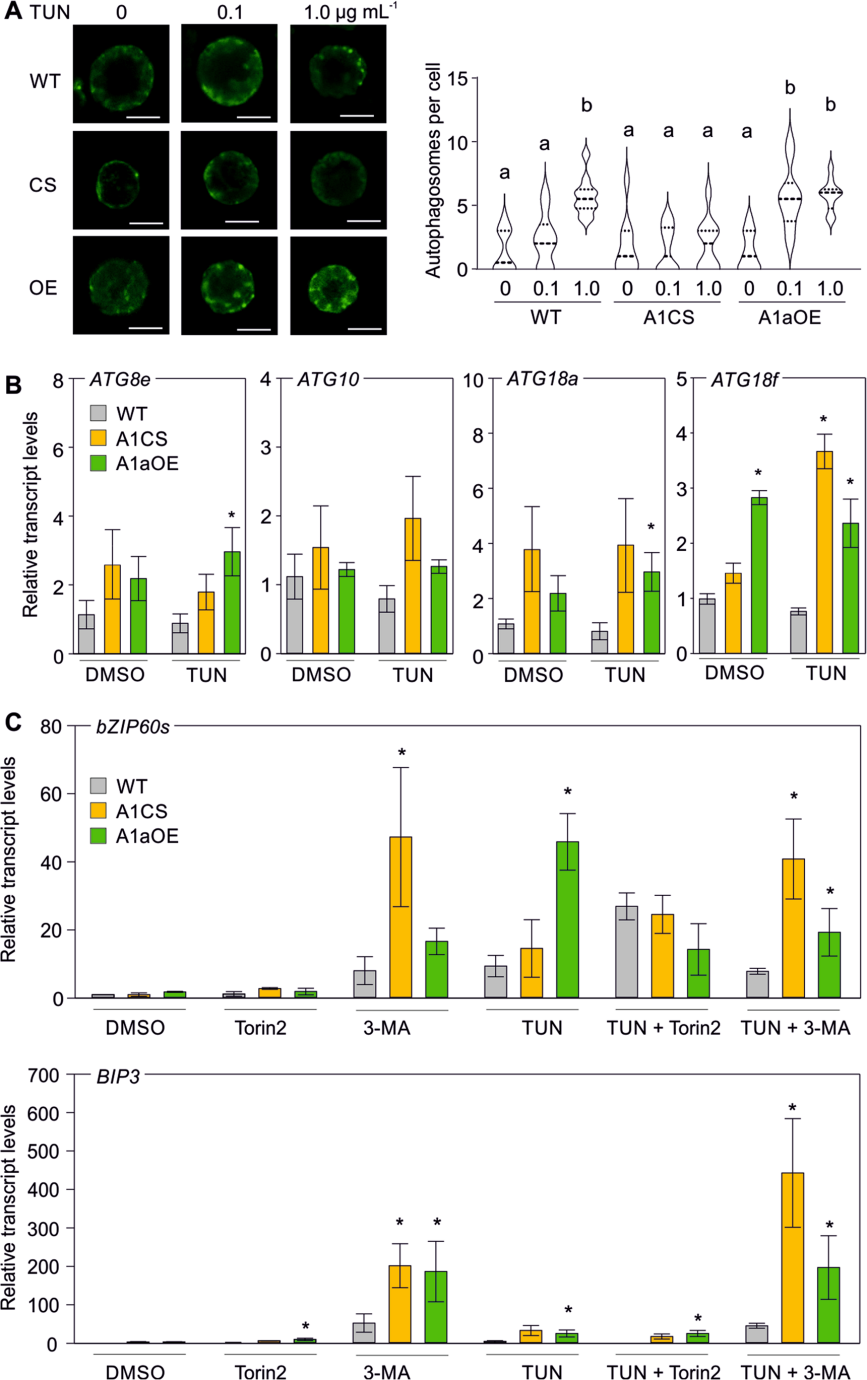
Regulation of ER stress–induced autophagy by HsfA1a. **A** Detection of MDC-stained autophagosomes of protoplasts from WT, A1CS, and A1aOE leaves treated with 0.1 or 1.0 μg mL^−1^ TUN or DMSO for 3 h. Bar is 10 μm. On the right, the number of autophagosomes detected in the same samples as in panel A is given. Violin plots from 10 cells for each sample. Different letters denote statistically significant differences based on ANOVA test and post hoc Duncan’s multiple range test (*p* < 0.05). **B** Transcript levels of ATG genes in WT, A1aOE, and A1CS treated with DMSO or TUN. **C** Transcript levels of BIP3 and bZIP60s in WT, A1CS, and A1aOE treated with DMSO or TUN as well as with 3-MA and Torin2. qRT-PCR values are the average of 3 biological replicates ± SEM. Asterisks denote statistically significant difference with the WT of the same treatment (*p < 0.05) based on *t*-test

ER stress induces autophagy as a protective mechanism against the accumulation of toxic protein aggregates (Liu et al. 2012). Considering that HS also induces autophagy in an HsfA1a-dependent manner (Xie et al. 2022), we examined the formation of autophagosomes in protoplasts isolated from leaves of WT, A1CS, and A1aOE plants using the MDC staining method (Bao et al. 2018). We observed a low number of autophagosomes in protoplasts of all genotypes treated with DMSO (Fig. 8A). The number of autophagosomes slightly increased when WT cells were treated with 100 ng mL^−1^ TUN and strongly elevated when treated with 1000 ng mL^−1^ (Fig. 8A). Such an increase did not occur in the A1CS cells, while A1aOE cells exhibited a dramatic increase in autophagosomes even under the milder TUN treatment.

This result raised the question whether HsfA1a exerts a transcriptional regulation of ATG genes. We examined the expression of nine ATG genes that are either HS-induced based on an in-house RNAseq (from leaves exposed to 40 vs 25 °C for 1 h; unpublished), or the presence of HSEs and regulation by HsfA1a either under drought or heat stress conditions as shown previously by other groups (Wang et al. 2015; Xie et al. 2022). We did not detect any significant induction of ATG genes in response to TUN treatment (Fig. 8B). However, *ATG18f* was expressed at higher levels in A1aOE leaves treated with DMSO and remained at increased levels compared to WT in TUN-treated samples (Fig. 8B). Interestingly, *ATG18f* was also expressed at higher levels in TUN-treated A1CS leaves treated with TUN. In addition, *ATG8e* and *ATG18a* showed increased levels after TUN treatment in A1aOE leaves when compared to corresponding WT sample (Fig. 8B). These results are in agreement with the stronger induction of autophagy in A1aOE plants which probably contributes to the increased tolerance of the transgenic seedlings to TUN treatment.

We also asked whether the enhanced or suppressed ER stress–induced autophagy in the A1aOE and A1CS cells compared to WT, is associated with the differences in UPR-genes in these lines. For this, autophagy was either blocked or enhanced with 3-MA and Torin2 treatments under control (DMSO) or ER stress–induced conditions (TUN). 3-MA is an inhibitor of phosphatidylinositol 3-kinases (PI3K) which are important for autophagosome formation (Seglen and Gordon 1982), while Torin2 (Montané and Menand 2013; Li et al. 2017) inhibits TOR and consequently induces autophagy (Pu et al. 2017). As a marker, we used *BIP3* which is regulated by bZIP60 and bZIP28 and its expression in response to TUN treatment is not altered by HsfA1a suppression or overexpression (Fig. 5B). In addition, we also monitored *bZIP60s* as a marker of ER proteotoxicity and IRE1 activity. The application of 3-MA resulted in higher levels of *bZIP60s* and *BIP3* in A1CS compared to WT both in the presence and absence of TUN, suggesting the inhibition of autophagy enhances ER-UPR response. *BIP3* was also expressed at higher levels in A1aOE leaves treated with Torin2 compared to WT (Fig. 8C). These results suggest that inhibition of TOR and concomitant ectopic expression of HsfA1a which both induce autophagy sensitize more UPR to ER stress.

## Discussion

Exposure of cells to high temperatures causes the accumulation of misfolded proteins. ER and cytosolic UPR mechanisms are well described but only recently the crosstalk between the two stress response pathways has been uncovered. In tomato, ER-UPR is mediated mainly by two transcription factors, bZIP28 and bZIP60. Our results confirm previous reports regarding the structure of the double stem loop and the predicted sites of RNA cleavage (Fig. 1). As shown previously for maize (Srivastava et al. 2018), the IRE1 inhibitor 4μ8c reduced the levels of bZIP60s, further supporting the conserved IRE1/bZIP60 pathway in tomato (Fig. 1). Furthermore, the GUS reporter assay indicates the direct regulation of several tomato genes that are induced by the ER stress elicitor TUN, including BIP and CNX-coding genes (Fig. 1).

bZIP28 in tomato is encoded by a single gene. The N-terminal region of the protein that is predicted to be exposed to the cytosol is the active transcription factor, as shown for its Arabidopsis orthologue. Interestingly, the N-terminal region of the protein is required for the transcriptional transactivation activity of the factor, but its presence leads to the high turnover of the protein (Fig. 2). Deletions of the parts of the N-terminal region reduced dramatically the transactivation activity of the factor but did not result in the stabilization of the protein. Interestingly, bZIP60s and bZIP28p showed similar activity on several GUS reporters, indicating that bZIP28 has very strong activity per se, and its high turnover is important for the control of the expression of ER-UPR genes. The induction of many GUS reporters by both bZIP28 and bZIP60 also indicates redundancy in regulation of ER-UPR genes, as previously shown in Arabidopsis (Figs. 1 and 2). However, a preferential regulation by one ER-UPR branch is also possible. For example, inhibition of IRE1 by 4μ8c in TUN-treated leaves resulted in reduced levels of BIP3 but not BIP2, suggesting a higher dependency of the former gene on the IRE1/bZIP60 pathway (Fig. 5). Of course, the contribution of other transcription factors cannot be excluded as well, including bZIP17 or NAC factors related to ER-UPR (Yang et al. 2014; Kim et al. 2018).

**Fig. 9:**
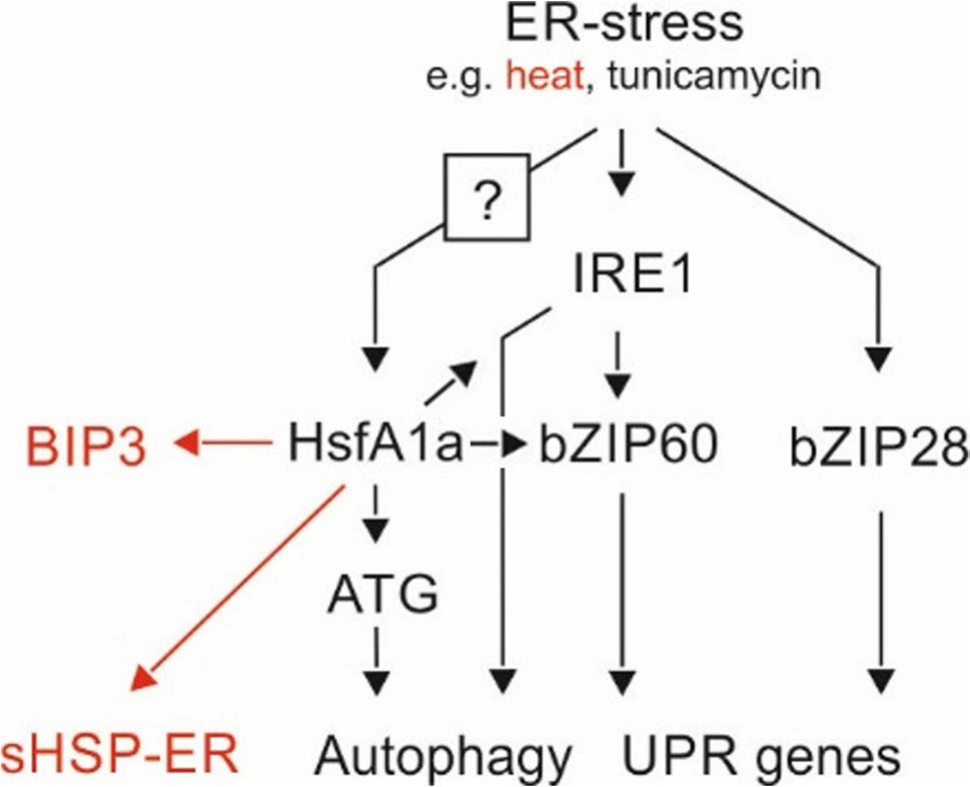
Hypothetical model for the regulation of ER-UPR by HsfA1a in tomato under proteotoxic conditions. bZIP60 and bZIP28 are the major and essential regulators of ER-UPR. Under heat stress, HsfA1a can regulate directly the transcription of ER sHSPs and co-regulate with bZIP60/bZIP28 the expression of BIP3. Through the control of ATG genes, HsfA1a can also regulate the ER stress–induced autophagy, a process that has been shown to be IRE1-dependent in *A. thaliana*. An active HsfA1a can also enhance the accumulation of bZIP60s, either by accumulation of bZIP60u and/or the increased activity of IRE1. The signal that triggers the activation of HsfA1a in response to ER stress is currently unknown and is marked with a question mark

ER-UPR genes are also HS-induced with most of them peaking after 1 h of stress at 40 °C (Fig. 3), as shown previously for the HSF-dependent HSFs and HSPs (Mesihovic et al. 2022). Several ER-UPR genes showed an altered expression in leaves of A1aOE and/or A1CS lines compared to WT. Therefore, the importance of HsfA1a is extended beyond the regulation of CPR, but also in the control of other UPR mechanisms as well. Remarkably, overexpression of HsfA1a resulted in an increased accumulation of both bZIP60u and bZIP60s (Fig. 3), indicating a regulation of bZIP60 transcription by HSFs or by transcription factors regulated by HSFs. This notion is supported by the high levels of *bZIP60s* in A1aOE leaves treated with both TUN and 4μ8c (Fig. 5). Alternatively, the mRNA of bZIPI60 might have also been stabilized in the A1aOE line compared to WT and/or the activity of IRE1 might be increased (Fig. 9).

The increased accumulation of *bZIP60s* in A1aOE plants is associated with the increased transcript levels of several ER-UPR genes, including *CNX2* and *BIP3* (Fig. 5). The latter also showed a dramatic induction in heat-stressed leaves of the A1aOE line (Fig. 3), suggesting that higher HsfA1a levels and proteotoxic conditions can have a dramatic stimulatory effect on the transcription of this gene, as under control conditions the induction was not as strong. The submergence of the whole seedling to TUN for 8 h resulted in increased hypocotyl elongation of A1CS seedlings compared to WT (Fig. 4). This is surprising considering that for example ER-sHSPs do not accumulate in A1CS. However, BIP2 was expressed at significantly higher levels in A1CS leaves exposed to DMSO compared to both DMSO treated WT and A1aOE, while Hsp90-7 was also induced in young leaves prior to HS. These results indicate that the A1CS tissues might by pre-acclimatized to ER stress (Fig. 5). This pre-acclimation might be due to the reduced levels of central players of the folding machinery in the ER, such as CNX2, which causes a weak activation of ER-UPR components. We have previously shown that some BIP proteins accumulate at higher levels in the leaves of a transgenic tomato line where HsfB1 is suppressed compared to WT (Fragkostefanakis et al. 2018). HsfB1 in this case could act as a repressor, as class B HSFs lack an activator domain. Considering that HsfB1 is an HsfA1a-dependent HS-induced gene, we can assume that a possibility for the induction of these genes in the A1CS line is the absence of HsfB1. However, this notion needs to be experimentally validated in the future.

An HSF-dependent regulation is reflected by the expression of the three ER-sHSPs which was reduced in A1CS and further increased in the A1aOE line under HS conditions compared to WT (Fig. 3). A positive and strong regulation of the three sHSPs by HSFs is also supported by the GUS reporter assay, where the activity of the HsfA1a-HsfA2 complex is apparent on these three promoters (Fig. 6). Their strong induction, similar to other cytosolic HSPs and HsfA1a-dependent HSFs in the early phase of the stress response, is essential for the protection of proteins against terminal aggregation (Waters and Vierling 2020). In Arabidopsis, the single ER-sHSP is not considered part of the ER-UPR as it is neither induced by ER stress elicitors, nor is it controlled by bZIP28 or bZIP60. However, bZIP60s stimulated the GUS activity of the PHsp21.5 GUS reporter while all three genes were induced by TUN (Figs. 1 and 3). Hsp21.5A is the only ER-sHSP coding gene to contain UPR *cis*-elements within the 500 nucleotide region upstream of the TSS (Fig. 6). Their upregulation in response to TUN could be due to the activation of HsfA1a, as GUS reporter assay with the strictly HSF-dependent Hsp17-CI promoter resulted in increased transactivation activity of HsfA1a. In tomato, Hsp21.5A is regulated by the transcription factor WHIRLY via an element which represents the binding motif “TFmatrixID_0865” corresponding to AtbZIP28. This indicates that bZIP28 and/or bZIP60 can also regulate Hsp21.5A. Indeed, co-expression of HsfA1a with bZIP28p or bZIP60s resulted in an approximately 4.5-fold induction in the GUS activity of the Hsp21.5A reporter, which however was not significantly based on the statistical analysis (Fig. 6).

The stronger accumulation of *bZIP60s*, *BIP3*, *CNX2*, and *sHSP* transcripts supports the increased tolerance of tomato seedlings to TUN treatment, providing clear evidence for a crosstalk between the two ER stress mechanisms (Fig. 5). While the tolerance against TUN in A1aOE line can be attributed to the increased accumulation of sHSPs, it is important to note the possibility of a direct co-regulation of some genes by both HSFs and bZIP60 and/or bZIP28. BIP3 promoter for example has both UPR and HS *cis*-elements in its promoter. Co-expression of either bZIP28p or bZIP60s with increasing amounts of HsfA1a/HsfA2 resulted in synergistic activity of the factors based on the GUS reporter assay (Fig. 7). This is in agreement with the very strong induction of BIP3 in the A1aOE line under HS. The presence of HSEs in promoter of genes is ubiquitous, and we confirmed this here for several ER-UPR genes (Fig. 6). However, the presence of an HSE does not necessarily mean that the gene is also regulated by HSFs, as in many cases neither HsfA1a nor the combination of HsfA1a/HsfA2 resulted in a significant induction of GUS activity in the corresponding reporter constructs (Fig. 6). Therefore, while we propose that notion of a co-regulation is probably valid, it should be revisited and validated further in the future. It should be noted as well that the accumulation of HsfA1a in some cases had a negative effect on transcript levels of some genes (Figs. 3 and 5). For example, *BIP1* and *Hsp90-7* were reduced in A1aOE line compared to WT, while *BIP2* was dramatically induced in the A1CS line.

Based on the proteostasis-based model for sensing HS, the activation of HsfA1a requires its release from the complex with cytosolic chaperones (Hahn et al. 2011). Interestingly, ER stress also results in a low activation of HsfA1a (Fig. 7). At stage, the signal that triggers this mild activation of HsfA1a is not known. However, the absence of induction of the HsfA2 and Hsp17.7A-CI indicates that this is not due to a sequential triggering of CPR. It is however possible that ER stress-related transcription factors induce other HSFs which could form heterooligomeric complexes with HsfA1a, or that the transgenic lines accumulate higher levels of co-activators or lower of co-repressors.

The increased tolerance of A1aOE seedlings in TUN treatment can be also explained by enhanced autophagy which can relieve proteotoxicity by removing deleterious protein aggregates. The more sensitized autophagy in A1aOE cells can be attributed to the increased expression of three ATG genes in A1aOE line (Fig. 8). In addition, autophagy can also affect ER-UPR activity as shown by the increased levels of BIP3 and bZIP60s in leaves treated with the autophagy inhibitor 3-MA. Interestingly, both HsfA1a overexpression and suppression sensitized more ER-UPR when autophagy was inhibited, suggesting a feedback mechanism between HsfA1a, autophagy, and ER-UPR. This is also supported by the lower accumulation of bZIP60s in TUN-treated leaves exposed to Torin2, a TOR inhibitor and thereby autophagy elicitor (Pu et al. 2017). Therefore, we propose that ER proteostasis is in part controlled by HSFs which regulate direct control of genes coding for ER-resident molecular chaperones and autophagy (Fig. 9).

## Conclusion

Our results provide evidence for the crosstalk between CPR and ER-UPR: the accumulation or suppression of HsfA1a is associated with altered levels of several ER-UPR genes which consequently leads to an increased resilience to ER stress. We propose four levels of regulation: (1) direct regulation of ER-UPR genes by HSFs, e.g., ER-sHSPs particularly under HS conditions; (2) regulation of bZIP60 transcription and/or splicing; (3) co-regulation of some ER-UPR genes by both bZIP60/bZIP28 and HSFs (e.g., BIP3); (4) regulation of ER stress–induced autophagy by HSFs (Fig. 9). The signal that triggers the activation of HsfA1a in response to ER stress is currently unknown. Additional experiments are required to validate into more detail this model.

## Supplementary Information

Below is the link to the electronic supplementary material.

Supplementary file1 (XLSX 27 KB)

Supplementary file2 (DOCX 1211 KB)

## Data Availability

All data generated or analyzed in this study are available from the corresponding author on reasonable request.
