# Supplementary material for: Crosstalk between endoplasmic reticulum and cytosolic unfolded protein response in tomato
Source: Cell Stress Chaperones. 2022 Nov 30;28(5):511–28. doi: 10.1007/s12192-022-01316-7 (PMC10469158; doi:10.1007/s12192-022-01316-7)
Supplement: Supplementary file 2 — Supplementary file2 (DOCX 1211 KB) [file 12192_2022_1316_MOESM2_ESM.docx]

## Supplementary Information for article

**Crosstalk between endoplasmic reticulum and cytosolic unfolded protein response in tomato**

^1^Karin Löchli, ^1^Emma Torbica, ^1^Misgana Haile-Weldeslasie, ^1^Deborah Baku, ^1^Aatika Aziz, ^1^Daniela Bublak, ^1,*^Sotirios Fragkostefanakis

^1^Molecular and Cell Biology of Plants, Goethe University Frankfurt, Germany D-60438

*Corresponding author: fragkost@bio.uni-frankfurt.de

**Supplementary Dataset 1.** *Cis*-elements identified in the promoters of ER-UPR genes (Excel file)

**Supplementary Figures**

**
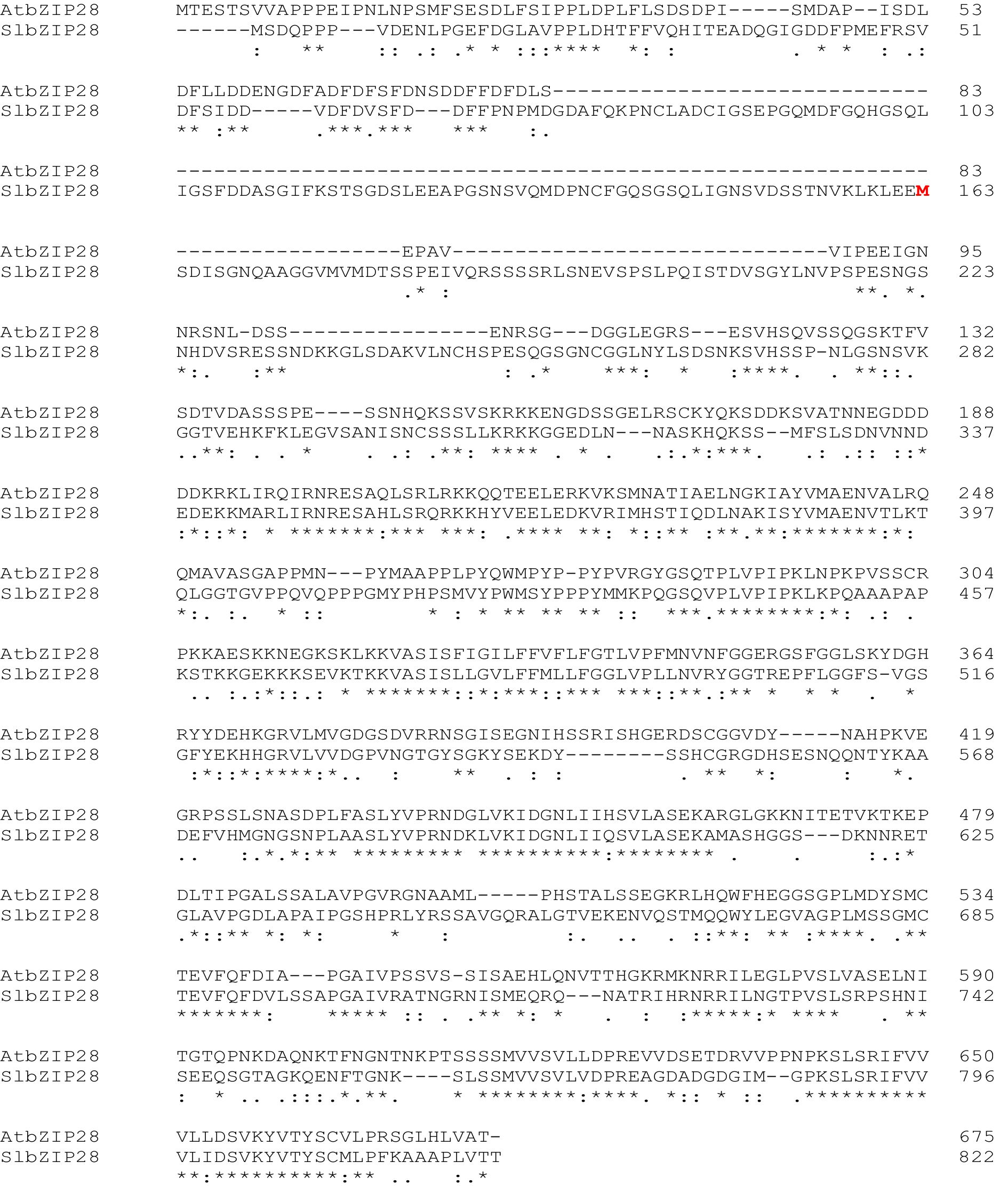
**

**Supplementary Figure 1.** Amino acid sequence alignment of A. thaliana and S. lycopersicum bZIP28 using Clustal Omega (ebi.ac.uk/Tools/msa/clustalo/)

**
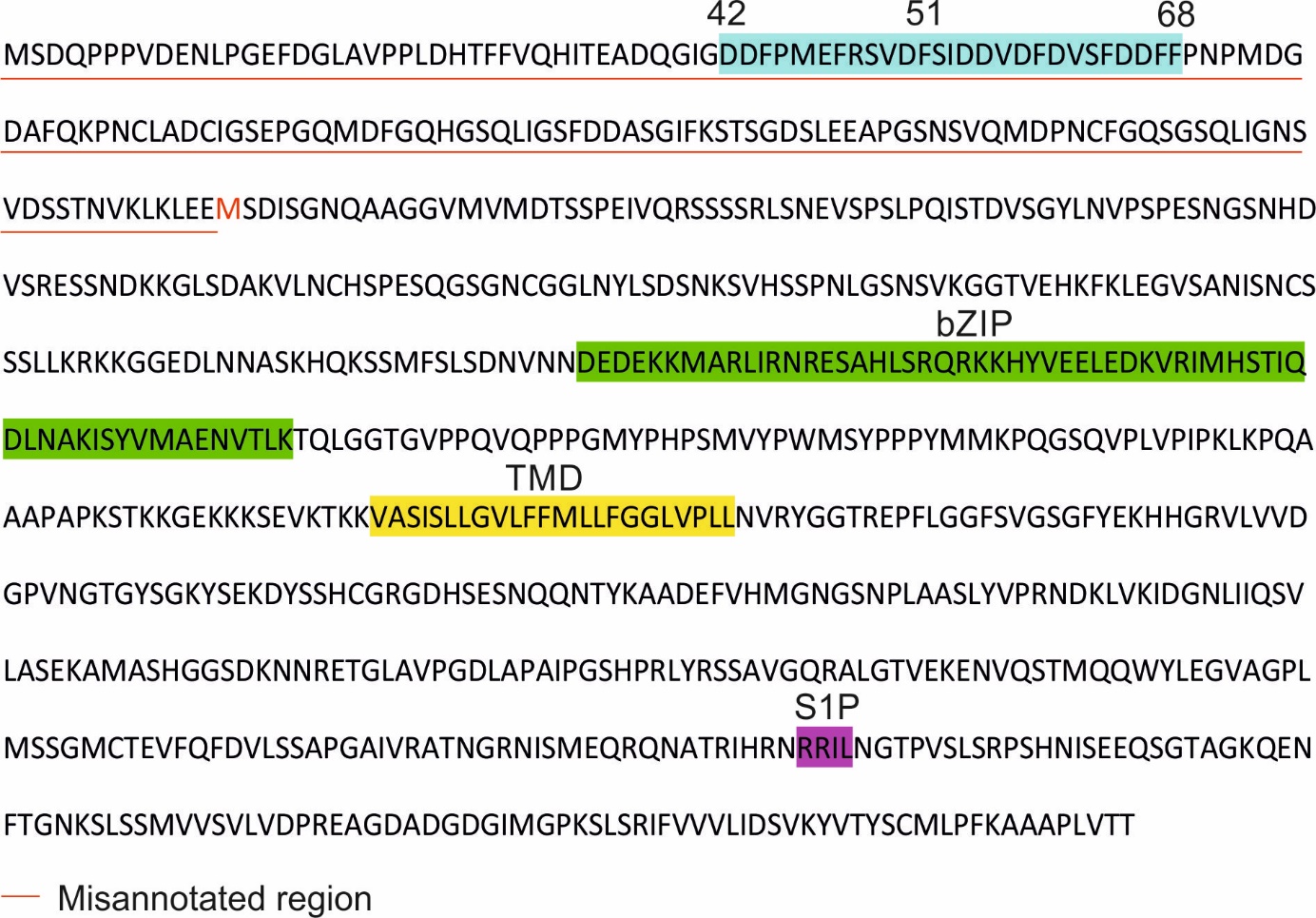
**

**Supplementary Figure 2.** Sequence and domains of tomato bZIP28

**
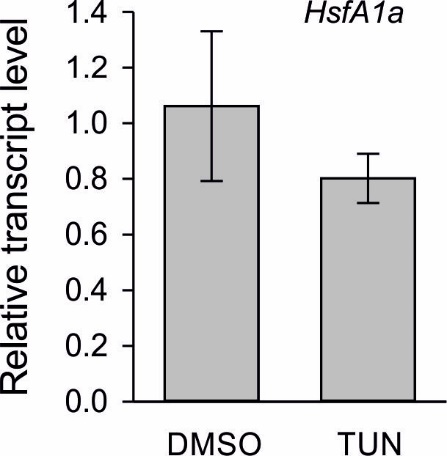
**

**Supplementary Figure 3.** Transcript levels of HsfA1a in WT plants treated with DMSO or TUN (50 ng mL^-1^) for 3 hours. Transcript levels were determined based on the 2-ΔΔCt method, with WT DMSO as control sample. Bars are the average of 3 independent replicates ±SEM. There is no statistical difference between the samples based on t-test (*p* < 0.05).


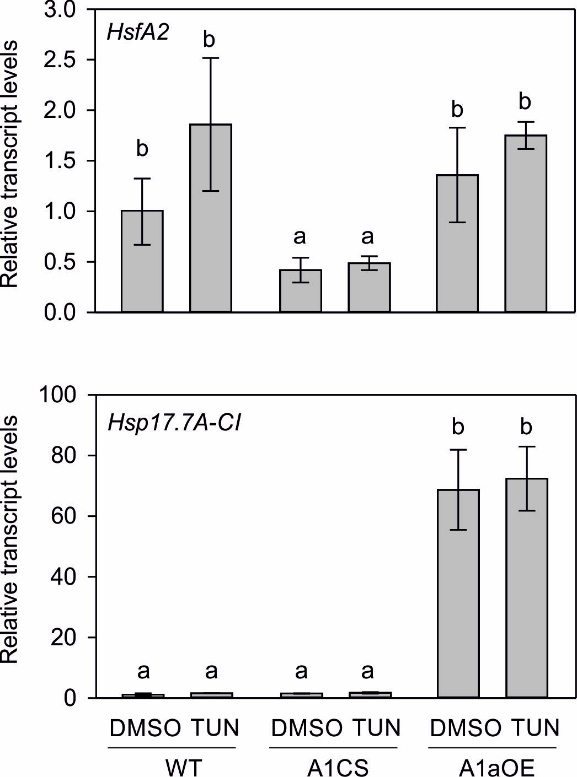


**Supplementary Figure 4.** Transcript levels of HsfA2 and Hsp17.7A-CI in leaves of WT, A1OE and A1CS treated with DMSO (0.1%) or TUN (50 ng mL^-1^) for 3 hours. Transcript levels were determined based on the 2^-ΔΔCt^ method, with WT DMSO as control sample. Bars are the average of 3 independent replicates ±SEM. Different letter denote statistically significant differences based on ANOVA and Duncan’s Multiple range test (*p* < 0.05).

**Supplementary Table 1.** Oligonucleotides used for qRT-PCR analysis.

| Gene name | Gene ID | Forward primer 5’->3’ | Reverse primer 5’->3’ |
| --- | --- | --- | --- |
| EF1a | Solyc06g009970 | GGAACTTGAGAAGGAGCCTAAG | CAACACCCACAGCAACAGTTT |
| bZIP60u | Solyc04g082890 | GGAAGAAGTTGTACGTTAGGGATCTTG | AGCAGGGATTCCAAAAAGAGCAC |
| bZIP60s | Solyc04g082890 | GGAAGAAGTTGTACGTTAGGGATCTTG | GCAGGGAACCCAACAGCTGAC |
| bZIP28 | Solyc10g078290 | AGAGACAGGCCTTGCAGTTC | GCACATTCTCCTTTTCTACAGTC |
| Hsp90-7 | Solyc04g081570 | CAGAGAAGGCCGAAGATGAGA | CGCCATGGGTTTATCTTCAC |
| BiP1 | Solyc08g082820 | TTGCAACCCAATCATCACAG | CCTCATTTCCTGCATCTTTGC |
| BiP2 | Solyc06g052050 | TGGTGGTGGAACATTTGATG | CATGCTTCTTCTTAATCACTCCATG |
| BiP3 | Solyc01g099660 | CAAAGTTGATTCCAAGAAACAGTAGG | GCGACAATCCTTCGTTAAGC |
| BiP4 | Solyc03g082920 | TGTTGGAGGAGTGATGACAAAG | CCCCAGTAGTCTACAGTCCTTTACC |
| Hsp21.5A | Solyc03g113930 | CCTGGAAATGCTGATTTGGA | AATATCCTCACCACCAACAGC |
| Hsp21.5 | Solyc11g020330 | ACTAAGCGCAGATGGGTCAT | CGCGAGCAGAGTGTTTTCTA |
| Hsp21.6 | Solyc01g102960 | AATTTGGATAATGGGGTGTTG | CAAATGATGATTATGATAAATAATCACA |
| CNX1 | Solyc03g118040 | GCTAGAGCTAGTGCCGAGACTG | TTCGACGAGGAGCAGCAC |
| CNX2 | Solyc06g068700 | CCGAGGAAGCAGCAAGCG | AAATGATAATTATGATTGAAATGACAG |

**Supplementary Table 2**. Oligonucleotides used for molecular cloning

| **Vector** | **Restriction sites** | **Oligonucleotide (5’ > 3’)** |
| --- | --- | --- |
| pRT-3HA-bZIP60s | SalI, BcuI | CAAGTCGACGATCGATAACATCGATGAT |
|  |  | TTGACTAGTCTAGTCTTTTTTCCCTCC |
| pRT-bZIP28(ΔN)-3HA | Acc65I-EcoRI | TTAGGTACCATGAGCGATATTTCTGGTAATC |
|  |  | TAAGAATTCTACCTCACTCTTCTTTTTCTC |
| pRT-ZIP28p-3HA | Acc65I, EcoRI | GACAGGTACCGATGTCCGATCAACCGCCAC |
|  |  | TAAGAATTCTACCTCACTCTTCTTTTTCTC |
| pRT-bZIP28pΔ53-68-3HA | Deletion PCR | TTCGGAGTGTAGATTTCCTAATCCTATGGATGGAGATGCG |
|  |  | CGATGGAATTTCGGAGTGTAGATTTCCTAATCCTATGGATG |
| pRT-bZIP28pΔ1-52-3HA | Deletion PCR | AGGTACCGATGTTCTCGATTGATGATGTTG |
|  |  | CATCAATCGAGAACATCGGTACCTGTCGACATG |
| pRT-bZIP28pΔ41-68-3HA | Deletion PCR | GGGATCGGTCCTAATCCTATGGATGGAG |
|  |  | TAGGATTAGGACCGATCCCCTGATCTGC |
| pRT-GFP-bZIP28p(ΔN) | Acc65I-BcuI | TTGGGTACCAATGAGCGATATTTCTGGTAATC |
|  |  | TTGACTAGTTACCTCACTCTTCTTTTTCTC |
| pRT-PBiP1::GUS | XhoI, SmiI | GACGATATCGAACAGACAGAATACACAAAAATTCAA |
|  |  | GACCTCGAGGATCTTACGCTCTGATTGGAG |
| pRT-PBiP3::GUS | NcoI,HindIII | CCCAAGCTTTATCTTGTTTTCACCAAATTGTG |
|  |  | CTAGTCTAGAAGCTGTTTCTTGCTTC |
| pRT-PHsp21.5A::GUS | HindIII, XbaI | CCCAAGCTTTCACATTAATTTAATTTTTAATG |
|  |  | CTAGTCTAGATTTGTTTGCTTCTATCAG |
| pRT-PHsp21.5::GUS | XhoI, HindIII | CCCAAGCTTTTCATCCTAATTTTACAG |
|  |  | CCGCTCGAGACTATACACTGTAGTATTG |
| pRT-PHsp21.6::GUS | HindIII, XbaI | CCCAAGCTTTATTCATTCTATGTTAGG |
|  |  | CTAGTCTAGAGCAGAGTTGGAGTTTATA |
| pRT-PCNX1::GUS | HindIII,XhoI | CCCAAGCTTATAGTGGGAAGCCAAGAC |
|  |  | CCGCTCGAGGAAATTAAAAGAAAAAATAGCA |
| pRT-PCNX2::GUS | HindIII, XbaI | CCCAAGCTTTCCACCATAAGAGTAAAG |
|  |  | CTAGTCTAGAATCGAGACATTCAAGAGTG |
